# Rhabdomyolysis Associated with Antimicrobial Drug–Resistant *Mycoplasma pneumoniae*

**DOI:** 10.3201/eid1805.111149

**Published:** 2012-05

**Authors:** Tomohiro Oishi, Mitsuo Narita, Hitomi Ohya, Takayuki Yamanaka, Yuta Aizawa, Mai Matsuo, Masamichi Matsunaga, Shinya Tsukano, Testuo Taguchi

**Affiliations:** Niigata University Medical and Dental Hospital, Niigata, Japan (T. Oishi);; Sapporo Tokushukai Hospital, Sapporo, Japan (M. Narita);; Kanagawa Prefectural Institute of Public Health, Kanagawa, Japan (H. Ohya);; Niigata Prefectural Shibata Hospital, Niigata (T. Yamanaka, Y. Aizawa, M. Matsuo, M. Matsunaga, S. Tsukano, T. Taguchi)

**Keywords:** ribosome, mutation, macrolide, bacteria, Japan, antimicrobial resistance, rhabdomyolysis, Mycoplasma pneumoniae

## Abstract

We describe a case of rhabdomyolysis in a patient infected with antimicrobial drug–resistant *Mycoplasma pneumoniae* The patient’s acute-phase serum levels of interleukin-18 and tumor necrosis factor–α were high, which suggests a pathogenic role for *M. pneumoniae*. In an era of increasing antimicrobial drug resistance, a system for rapidly identifying resistant *M. pneumoniae* would be beneficial.

*Mycoplasma pneumoniae*, one of the major causes of community-acquired pneumonia in children, can cause a variety of extrapulmonary manifestations. Antimicrobial drug–resistant strains of *M. pneumoniae* have been isolated from children in Japan; however, to our knowledge, extrapulmonary manifestations caused by antimicrobial drug–resistant *M. pneumoniae* have not been reported. We describe a case of rhabdomyolysis, the rapid breakdown of striated muscle, in a 7-year-old girl in Japan who had antimicrobial drug–resistant *M. pneumoniae* infection, and we discuss the possible pathomechanisms for rhabdomyolysis.

## Case Report

A 7-year-old girl had been healthy until 7 days before she was admitted to Niigata Prefectural Hospital, Niigata, Japan, on June 21, 2010, for cough and prolonged fever. On day 1 of her illness, the girl had visited her primary care physician and was prescribed azithromycin, a macrolide antimicrobial drug, for a lower respiratory tract infection. On day 7 of her illness, the girl’s condition worsened acutely, with increased cough and fever, and she again visited her primary care physician. A chest radiograph showed pulmonary infiltrates in the left upper lung, and the patient was referred to our hospital on day 8 of her illness. The girl’s history and family history were unremarkable.

On hospital admission, the patient was alert and oriented. Her temperature was 38.9°C, heart rate was 101 beats/min, and oxygen saturation was 97%. Chest auscultation was unremarkable. The girl did not describe symptoms of myalgia, and physical examination did not show signs of erythema, hepatosplenomegaly, neurologic abnormalities, muscle weakness, or muscle atrophy.

Results of the initial laboratory test were as follows: leukocyte count, 6.2 × 10^9^ cells/L (reference 3.0–8.6 × 10^9^ cells/L); hemoglobin, 1.95 mmol/L (reference 1.67–2.31 mmol/L); platelet count, 23.3 × 10^9^/L (reference 15.0–36.1 × 10^9^/L); C-reactive protein, 27 mg/L (reference <3.0 mg/L); aspartate aminotransferase, 161 IU/L (reference 13–31 IU/L); alanine aminotransferase, 83 IU/L (reference 6–27 IU/L); lactate dehydrogenase, 691 IU/L (reference119–229 IU/L); blood urea nitrogen, 3.2 mmol urea/L (reference 2.9–7.2 mmol urea/L); creatinine, 31.8 μmol/L (reference 44.2–70.6 μmol/L); sodium, 135 mmol/L (reference 138–146 mmol/L); potassium, 4.1 mmol/L (reference 3.6–4.9 mmol/L); and chloride 96 mmol/L (reference 99–109 mmol/L). A venous blood gas determination on room air showed a pH of 7.464 (reference 7.35–7.45kPa) and carbon dioxide partial pressure of 4.9 kPa (reference 4.7–6.0 kPa). Levels of serum glucose, albumin, calcium, amylase, and bilirubin were normal (references 70–109 mg/dL, 4.1–5.0 g/dL, 8.7–10.0 mg/dL, 39–108 U/mL, and 0.3–0.9 mg/dL, respectively). Creatine phosphokinase was elevated to 12,159 ng/mL (reference 45–163 ng/mL). Urinalysis showed blood 3+, but analysis of urine sediment by microscopy showed no erythrocytes. The urine myoglobin level was 39,900 μg/L (reference <10 μg/L). No antinuclear factor or circulating immune complex was detected. The serum concentration of cytokine interleukin (IL)-18 on admission was 612 pg/mL (reference <260 pg/mL), and the concentration of tumor necrosis factor–α (TNF-α) was 3.48 pg/mL (reference <1.79 pg/mL).

The girl’s fever did not respond to treatment with azithromycin, and she was given a tentative diagnosis of antimicrobial drug–resistant *M. pneumoniae* infection, which was prevalent in the region. Because the patient was <8 years of age, she was started on treatment with tosufloxacin, a fluoroquinolone, granules for children (12 mg/kg/d) and steroid therapy (methylprednisolone, 1 mg/kg/d). The patient’s fever resolved the next day, and her urine output was maintained with intravenous hydration. No signs or symptoms of muscle involvement developed during the patient’s hospital stay.

On day 16 after the patient was admitted to the hospital, results of laboratory testing showed improved values for creatine phosphokinase (1,855 ng/mL), aspartate aminotransferase (101 IU/L), alanine aminotransferase (162 IU/L), lactate dehydrogenase (294 IU/L), and urine myoglobulin (10 μg/L). Pulmonary infiltrates seen on a chest radiograph had decreased substantially by day 16, and the patient was discharged from the hospital. On day 8 after discharge, her abnormal test results returned to normal, and her illness showed no signs of relapse.

Culture results for a respiratory sample obtained during hospitalization revealed normal bacterial flora, and the results for rapid diagnostic tests for influenza virus, adenovirus, and respiratory syncytial virus were negative at admission. The *M. pneumoniae* antibody titer by the particle agglutination test was 1,280 at admission, and 2 days later, the antibody titer had increased to 10,240. At hospital admission, with permission from the girl and her parents, a pharyngeal swab specimen was obtained to test for *M. pneumoniae*. The sample was sent to the laboratory of the Kanagawa Prefectural Institute of Public Health, Chigasaki, Japan, where PCR and restriction fragment length polymorphism analysis were performed as described (*1*). A macrolide-resistant *M. pneumoniae* strain with an A→G transition at position 2063 of the 23S rRNA gene (designated A2063G) was detected. Laboratory test results from admission to 8 days after discharge are summarized in the Table.

**Table:** Laboratory test results for a 7-year-old patient with rhabdomyolysis associated with antimicrobial drug–resistant *Mycoplasma pneumonia*e, Japan, June 22–July 23, 2010*

Laboratory test	Laboratory value, by days after onset of fever					
	7 d	9 d	13 d	17 d	23 d	31 d
Leukocytes, ×10^9^ cells/L	6.2	8.0	11.8	7.3	5.9	4.7
C-reactive protein, mg/L	27	7	4	1	<1	<1
Creatine phosphokinase, mg/L	12,159	12,918	10,937	5,839	1,855	130
Aspartate aminotransferase, IU/L	161	203	169	94	101	24
Alanine aminotransferase, IU/L	83	205	284	209	162	37
Lactate dehydrogenase, IU/L	691	553	451	358	294	234
Creatinine, μmol/L	31.8	23.0	30.1	32.7	30.9	29.2
Urine myoglobin, μg/L	39,900	NT	2,500	NT	10	<10
Urine erythrocytes	3+	3+	±	−	−	−
Serum interleukin-18, pg/mL	612	519	NT	NT	367	232
Serum tumor necrosis factor–α, pg/mL	3.48	3.38	NT	NT	2.03	1.64
*M. pneumoniae* titer	1,280	10,240	NT	NT	NT	NT

*NT, not tested.

## Conclusions

This case of rhabdomyolysis in a 7-year-old girl is an unusual extrapulmonary manifestation of antimicrobial drug–resistant *M. pneumoniae* infection. Rhabdomyolysis is characterized by rupture and necrosis of muscle fibers, resulting in the release of cell breakdown products into the bloodstream and extracellular space. Direct muscle injury is the most common cause of rhabdomyolysis, but a number of other causes are possible: hereditary enzyme disorders, drugs, toxins, endocrinopathies, malignant hyperthermia, neuroleptic malignant syndrome, heatstroke, hypothermia, electrolyte alterations, diabetic ketoacidosis and nonketotic hyperosmolar coma, severe hypothyroidism or hyperthyroidism, and bacterial or viral infections (*2*). Bacterial and viral infections account for ≈5% of rhabdomyolysis cases in adults (*3*).

Because pathomechanisms other than infection can cause rhabdomyolysis (*4**,**5*), we cannot say with certainty that *M. pneumoniae* infection caused this syndrome in the patient reported here. One possible mechanism of rhabdomyolysis is induction of inflammatory cytokines, such as TNF-α and IL-1. These cytokines can cause acute proteolysis in a variety of organs, including skeletal muscles (*6**,**7*), and *M. pneumoniae* can induce these cytokines (*8*). Our patient had high levels of TNF-α and IL-18 during the acute phase of *M. pneumoniae* infection, and it is highly possible that these *M. pneumoniae*–induced cytokines were involved in the pathomechanism of rhabdomyolysis. No other apparent cause, such as trauma, endocrine disorder, or infection, other than *M. pneumoniae*, was found for the development of rhabdomyolysis in this patient.

A confounding factor in this case was that the extrapulmonary manifestation of *M. pneumoniae* infection was caused by an antimicrobial drug–resistant strain of *M. pneumoniae*. The implicated strain, A2063G, is the dominant type of antimicrobial drug–resistant *M. pneumoniae* in Japan (*1**,**9**,**10*). Approximately 15% of *M. pneumoniae* strains isolated from patients in Japan are resistant to antimicrobial drugs (*1**,**9**,**10*), which may explain why extrapulmonary manifestations of antimicrobial drug–resistant *M. pneumoniae* have not been frequently reported. However, the proportion of antimicrobial drug–resistance in Japan is increasing, so extrapulmonary manifestations of antimicrobial drug–resistant *M. pneumoniae* infection might also increase.

In conclusion, this case of rhabdomyolysis was associated with and, in the absence of any other apparent cause, appears to be attributable to infection with antimicrobial drug–resistant *M. pneumoniae*. The development of a system that can be used in routine clinical practice to rapidly identify antimicrobial drug–resistant *M. pneumoniae* would be highly beneficial in this era of increasing antimicrobial drug resistance.
